# Emerging technologies in citizen science and potential for insect monitoring

**DOI:** 10.1098/rstb.2023.0106

**Published:** 2024-06-24

**Authors:** Julie Koch Sheard, Tim Adriaens, Diana E. Bowler, Andrea Büermann, Corey T. Callaghan, Elodie C. M. Camprasse, Shawan Chowdhury, Thore Engel, Elizabeth A. Finch, Julia von Gönner, Pen-Yuan Hsing, Peter Mikula, Rui Ying Rachel Oh, Birte Peters, Shyam S. Phartyal, Michael J. O. Pocock, Jana Wäldchen, Aletta Bonn

**Affiliations:** ^1^ Department of Ecosystem Services, Helmholtz Centre for Environmental Research - UFZ, Permoserstraße 15, 04318 Leipzig, Germany; ^2^ Institute of Biodiversity, Friedrich Schiller University Jena, Dornburger Straße 159, 07743 Jena, Germany; ^3^ German Centre for Integrative Biodiversity Research (iDiv) Halle-Jena-Leipzig, Puschstraße 4, 04103 Leipzig, Germany; ^4^ Research Institute for Nature and Forest (INBO), Havenlaan 88 bus 73, 1000 Brussels, Belgium; ^5^ UK Centre for Ecology & Hydrology, Wallingford, Oxfordshire, OX10 8BB, UK; ^6^ Department of Wildlife Ecology and Conservation, Fort Lauderdale Research and Education Center, University of Florida, FL 33314, USA; ^7^ School of Life and Environmental Sciences, Deakin University, Melbourne Burwood Campus, 221 Burwood Highway, Burwood, Victoria 3125, Australia; ^8^ Faculty of Life Sciences, University of Bristol, 12a Priory Road, Bristol BS8 1TU, UK; ^9^ TUM School of Life Sciences, Ecoclimatology, Technical University of Munich, Hans-Carl-von-Carlowitz-Platz 2, 85354 Freising, Germany; ^10^ Institute for Advanced Study, Technical University of Munich, Lichtenbergstraße 2a, 85748 Garching, Germany; ^11^ Faculty of Environmental Sciences, Czech University of Life Sciences Prague, Kamýcká 129, 16500 Prague, Czech Republic; ^12^ School of Ecology and Environment Studies, Nalanda University, Rajgir 803116, India; ^13^ Department of Biogeochemical Integration, Max Planck Institute for Biogeochemistry, Hans-Knöll-Straße 10, 07745 Jena, Germany

**Keywords:** biodiversity monitoring, community science, novel technologies, public participation in scientific research, insects, artificial intelligence

## Abstract

Emerging technologies are increasingly employed in environmental citizen science projects. This integration offers benefits and opportunities for scientists and participants alike. Citizen science can support large-scale, long-term monitoring of species occurrences, behaviour and interactions. At the same time, technologies can foster participant engagement, regardless of pre-existing taxonomic expertise or experience, and permit new types of data to be collected. Yet, technologies may also create challenges by potentially increasing financial costs, necessitating technological expertise or demanding training of participants. Technology could also reduce people's direct involvement and engagement with nature. In this perspective, we discuss how current technologies have spurred an increase in citizen science projects and how the implementation of emerging technologies in citizen science may enhance scientific impact and public engagement. We show how technology can act as (i) a facilitator of current citizen science and monitoring efforts, (ii) an enabler of new research opportunities, and (iii) a transformer of science, policy and public participation, but could also become (iv) an inhibitor of participation, equity and scientific rigour. Technology is developing fast and promises to provide many exciting opportunities for citizen science and insect monitoring, but while we seize these opportunities, we must remain vigilant against potential risks.

This article is part of the theme issue ‘Towards a toolkit for global insect biodiversity monitoring’.

## 1. Introduction

**Figure 1.**
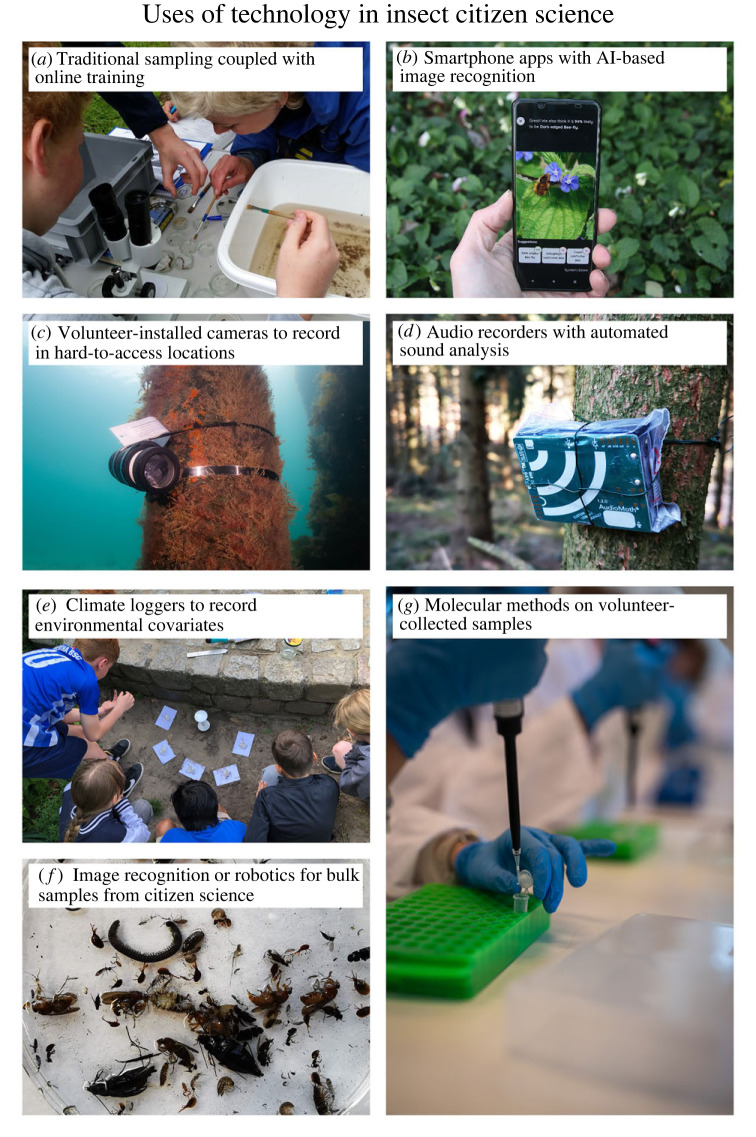
Technology is developing fast and promises to provide many exciting opportunities for citizen science and insect monitoring, including (*a*) online training of volunteers; as in the German FLOW project (photo by Julia von Gönner), (*b*) smartphone apps with image recognition to verify identifications submitted by recorders; as demonstrated in the iRecord smartphone app (photo by Michael Pocock), (*c*) cameras for detecting species in hard-to-access locations and during unsociable hours; as demonstrated by the Australian Spider Crab Watch project (photo by Elodie Camprasse), (*d*) audio recorders combined with automated sound analysis for vocalizing species; here an AudioMoth recorder from Open Acoustic Devices (photo by Julie K. Sheard), (*e*) climate loggers for recording environmental covariates; as used in the German MikroSafari project (photo by Aletta Bonn), (*f*) image recognition or robotics for bulk samples (photo by Julie K. Sheard) and (*g*) molecular methods on volunteer-collected samples where laboratory work is also carried out by volunteers; as demonstrated by the Danish DNA&Liv project (photo by Frederik Wolff Nisbeth Teglhus).

Citizen science, also referred to as community science or public participation in scientific research, is a practice with historical roots dating back centuries. This collaborative approach to scientific investigation involves individuals from diverse backgrounds, including those without formal scientific training, actively engaging in research activities [1,2]. The number and diversity of citizen science projects and their significance have grown in the twenty-first century, thanks in large part to advancements in technology, including the widespread availability of the Internet and the proliferation of digital platforms [3–6]. Today, technological equipment, such as mobile phones and digital cameras, along with their applications (e.g. smartphone apps), are commonly used in citizen science projects, potentially shifting project design towards simpler, mass-participation approaches [4] (figure 1).

Aided by an open survey and two online workshops, we discuss how the rapid advancement of current technologies has spurred an increase in citizen science projects. We highlight how the implementation of emerging technologies—technologies in the early stages of development, adoption and commercialization—may act as facilitators of what is already being done, enablers of new research avenues, transformers of science, policy and public participation and/or inhibitors of equity and openness in citizen science biodiversity research.

As an incredibly diverse and abundant animal group [7], insects play major roles in ecosystem functioning [8], yet despite widespread concern about their decline [8,9], our understanding of their conservation status is limited [10,11]. For this special issue, we focus on possible ways to improve insect monitoring techniques through the integration of technology in citizen science projects, but we note that our perspective is broadly relevant to biodiversity monitoring beyond insects.

## 2. The rapid advancement of technology has spurred an increase in citizen science projects

Smartphone applications have already revolutionized data collection and submission of species observations [4,12]. The Global Biodiversity Information Facility (GBIF) is the largest aggregator of biodiversity records in the world, where species occurrence data are primarily sourced through disparate citizen science applications. It is estimated that in 2020, as much as 65% of all data on GBIF [13] and a minimum of 74.5% of all insect observations [14] were contributed by citizen scientists.

Technology, including instant messaging apps, social media and online meeting tools, has revolutionized data transfer and strengthened connections among participants, project managers and researchers across vast distances. This has promoted engagement and facilitated the exchange of training and data (Box 1). The simplicity of creating short training videos, direct interaction with fellow citizen scientists and self-organized support through social media networks foster a sense of community and enrich opportunities for collaborative research and exchange [18]. Social media are also being harnessed to address biodiversity data gaps, as many people use these channels to share species photographs [19–21]. Besides smartphone applications and social media, other emerging technologies are currently being developed to assist with biodiversity monitoring. These include, but are not limited to, computer vision, acoustic monitoring, radar and molecular methods [22]. These technologies are also being included, to varying extents, in citizen science projects (figure 1), but the extent of uptake and experiences of application have yet to be documented.

Box 1. **The potential of citizen science to advance insect research – Project FLOW.**

The citizen science project FLOW (www.flow-projekt.de) invites citizen groups (e.g. high school students, members of fishing clubs and environmental NGOs) to analyse the ecological status and pesticide exposure of their local streams by sampling benthic invertebrates [15,16]. Participants are trained through online identification guides, video tutorials and online quizzes along with yearly online and on-site training sessions, which are important because learning to identify benthic invertebrates requires hands-on practice and personal, direct feedback from experts.

By providing an engaging approach to insect monitoring and identification, citizen science projects such as FLOW can help increase public awareness of insect diversity, and particularly of lesser-studied taxonomic groups [17]. Thus, citizen scientists can help increase the availability of data on underrepresented insect taxa such as caddisflies, mayflies or stoneflies. In addition, experienced citizen scientists can participate in the analysis and digitization of insect data. This citizen engagement can be strongly supported by digital data management tools. For example, the FLOW project provides a web application for collecting, analysing, visualizing, accessing and archiving citizen science data, which is integrated into the coordinating institute's biodiversity data platform. The web application allows project coordinators and external experts to assess data quality, e.g. by reviewing photo vouchers of assessed stream sites and identified species. Potential data users can use the web application to request access to the FLOW data.

The development and launch of the FLOW web application, however, also presented challenges. To successfully establish a new digital citizen science data management system, it is important to clearly identify the goals, functions and working methods of the new tool and communicate them in an easy to understand and engaging way to the volunteers. Importantly, at the point of development, different user perspectives and user feedback should be included in the technical development process. This needs to be integrated from the beginning of the project to create acceptance for the new digital system and to integrate it permanently into the research activities of citizen scientists.

Furthermore, successful implementation of digital data management requires a certain level of media literacy and understanding on the part of the volunteers. As these skills often vary widely across the community, support services such as instructional videos, wikis/FAQs or personalized email/phone consultations are helpful in enabling different audiences to access and use the digital tools.

By addressing these challenges and creating synergies between participatory insect monitoring and digital data management, citizen science projects such as FLOW can help produce valuable insect data and reduce taxonomic bias, thereby advancing insect research.

## 3. A survey of current use of technology in citizen science

We conducted an open online survey from 9th July to 14th August 2023 that was distributed via email and social media. The target audience included anyone with experience in citizen science, whether as a project coordinator or participant, and regardless of whether or not they used technology. The survey was focused towards biodiversity citizen science projects, but was kept intentionally broad to ensure that we captured as comprehensive a spectrum of technologies and applications as possible, such that we could then explore and expand the use of technologies not currently used for insect citizen science projects. The questionnaire was approved as anonymous and performed in accordance with relevant guidelines and regulation according to the legal department of the Helmholtz-Zentrum für Umweltforschung - UFZ and disseminated using the open source web application LimeSurvey [23]. Informed consent was obtained from all respondents. Further methodology and the full survey have been uploaded to Zenodo [24].

A total of 70 respondents from Europe (40 respondents), North America (12 respondents), Asia (7 respondents), Oceania/Australia (6 respondents) and Africa (5 respondents), representing 66 citizen science projects and platforms, completed the full survey [24]. Most respondents were citizen science project leaders, organizers or coordinators (55 respondents), of which 23 respondents had more than 10 years of experience working with citizen science.

**Figure 2.**
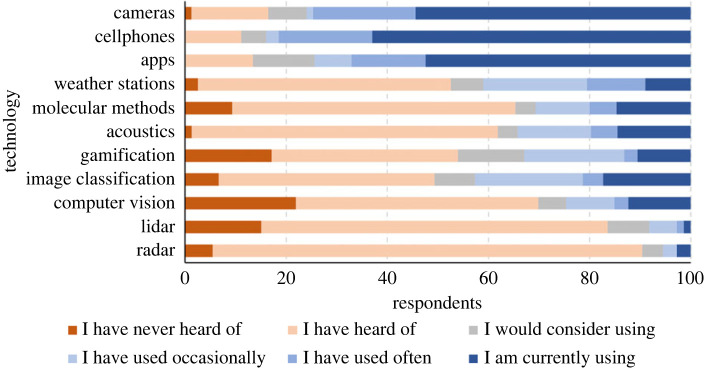
Online survey responses from 70 participants to the question ‘Please indicate which of the following technologies you are familiar with and how. Check all that apply’. This led to some categories with over 70 responses. While cameras, cell phones and apps are widely used in citizen science projects, more advanced and emerging technologies have seen less uptake. Technologies are ordered by the response ‘I have used often’.

The survey focused largely on how technology is acting as a facilitator for current citizen science projects and highlighted 11 example technologies and applications that are used in citizen science projects (figure 2). Respondents stated that cameras, smartphones and apps were the most commonly used of the 11 technologies. Other studies have considered an even wider range of technologies that are, or could be, implemented in citizen science (see for example [25]).

Overall, there was a positive attitude among survey respondents towards the inclusion of technologies in citizen science projects (electronic supplementary material, figure S1, Q1–3), with 91% agreeing or strongly agreeing that they will use technologies for citizen science in the future and 86% agreeing or strongly agreeing that using technologies will benefit citizen science by increasing the data quality and impact (electronic supplementary material, figure S1, Q6+7). There was also a strong belief among these respondents that technologies make it easier to participate in citizen science and increase learning (electronic supplementary material, figure S1, Q5+8). Despite this, respondents felt that implementing technologies will not necessarily increase the attractiveness of participating, with only 44% agreeing or strongly agreeing that implementing technologies in citizen science would increase their willingness to participate (electronic supplementary material, figure S1, Q9). Nor would technology necessarily help participants be more engaged in nature, with 43% agreeing or strongly agreeing that the use of technology would help them be more engaged in nature (electronic supplementary material, figure S1, Q10). Another caution was that only 41% of the respondents agreed or strongly agreed that it is easy to learn to use technologies for citizen science, and 40% agreed or strongly agreed that it is easy to become skilful in using technologies (electronic supplementary material figure s1, Q11–12).

**Figure 3.**
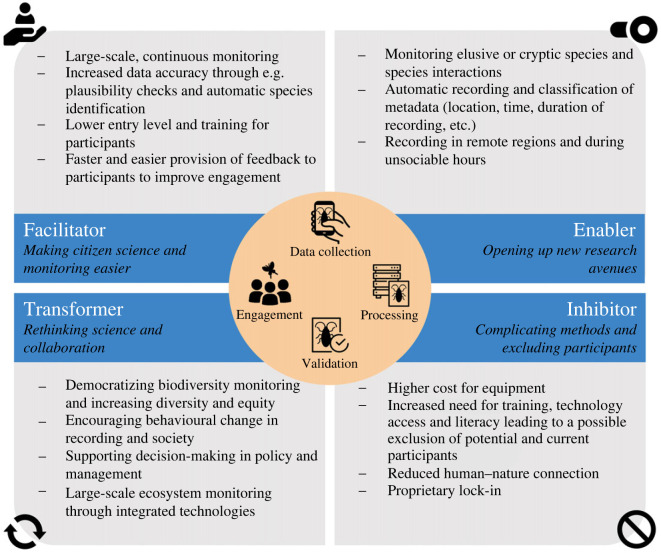
Technology as an enabler, facilitator, transformer and inhibitor of citizen science.

Two follow-up online workshops held on 30th and 31st August 2023 with 15 participants aimed to elicit further in-depth discussions and reflections on how the technologies could affect citizen science initiatives and enable us to pursue new research avenues and transform how we do citizen science for insect monitoring in the future. Most of the workshop participants joined in writing this paper and as a result of these insights, we frame the rest of the paper around four themes, namely (i) Technology as a facilitator—making citizen science and monitoring easier, (ii) Technology as an enabler—opening up new research avenues, (iii) Technology as a transformer—rethinking science and collaboration and (iv) Technology as an inhibitor—complicating methods and excluding participants (figure 3). We keep our discussions broad to consider opportunities and challenges of technology for citizen science in general, and conclude with a perspective for future directions for insect monitoring. A list of all citizen science projects, apps and platforms mentioned in this paper has been included as electronic supplementary material S2.

## 4. Technology as a facilitator—making citizen science and monitoring easier

The integration of technology in citizen science has already proven very useful in facilitating existing citizen science projects through improved data collection, identification and verification [4,12], along with the collection of more detailed environmental and ecological data. In particular, smartphone apps have increased the quality of data by facilitating metadata collection, such as automatically recording location through integrated global positioning systems (GPS) and date and time information [26–28]. Furthermore, using smartphone apps for live-tracking of location and time spent on a citizen science activity captures information on observation effort, which is a crucial variable to accurately estimate species occurrence trends and/or compare data from atlases and checklists.

Apps and technology have also been developed to help people collect more detailed ecological data. For instance, some citizen science projects have been developed to study plant–pollinator interactions, by asking participants to spend 15–20 min photographing every invertebrate landing on a focal flower [29,30]. Continuous monitoring of ecosystem services, such as pollination, has historically been labour-intensive and is rarely done to species level [31], but the development of camera traps for automated pollinator monitoring with built-in insect classification is revolutionizing this [32,33]. Open access, DIY (do it yourself) instructions are now available that enable anyone to build or adapt camera traps or photomicroscopes for insect monitoring, including hardware assembly, software setup, programming, model training and deployment (e.g. [34,35]). Utilizing technology to perform the labour-intensive job of continuous recording opens the possibility for participants to focus on the enjoyment of observing species. However, for some participants, the use of DIY technology may also represent motivation in itself [28,36]. These participants could be aided by citizen science platforms, such as SciStarter, which is compiling a database of tools that citizen scientists and project coordinators can build, borrow or buy [36]. Today, many people have personal weather stations in their homes. Crowdsourcing information from these stations can provide detailed information on climatic conditions, especially in urban areas [37,38]. Since most insect species live either on or near the soil surface, they are very sensitive to changes in climatic conditions, so the use of citizen science to collect microclimate data (soil temperature and moisture) through weather stations or projects like SoilTemp [39] combined with macroclimate (wet/dry and cold/warm season) data can help us model the environmental niches of insect species or predict insect pest populations and their control (e.g. [40]).

In terms of data processing and species identification, automated image recognition and sound classification used in apps such as Seek, eButterfly, Flora Incognita, Pl@ntNet, ObsIdentify and BirdNET, have opened up the identification of nature to everyone, helping participants to verify their observations regardless of their identification skills [41–43]. One example is the Mosquito Alert citizen science system, which incorporates a dedicated mobile application for the collection of geotagged images. This system offers a practical means of tracking the global distribution of mosquito species, although its efficacy depends upon the quality of the submitted photos. Expert entomologists review the submitted images, so providing feedback to the participating volunteers and generating verified data that are valuable for public health agencies. Automated identification tools can also enable the collection of secondary data, such as species interactions, from submitted records [44]. For instance, plants could be automatically identified from photos of flower-visiting insects submitted by citizen scientists to capture these plant–pollinator interactions.

As data amounts increase, relying on manual inspection for species identification based on images or sounds is slow, laboursome and not a sustainable long-term solution. Recordings made and classified by citizen scientists are simultaneously playing a crucial role in developing automated methods. Citizen science apps are helping to build training datasets for the development of classification algorithms [45–48]. In the Mosquito Alert project, a deep-learning model was trained on the curated image library to detect tiger mosquitoes (*Aedes albopictus*), an invasive species responsible for transmitting diseases like chikungunya, dengue and Zika [49]. InsectNet is proposing a deep-learning model that will be trained on data from iNaturalist and capable of robustly identifying insect species in images with even the most complex backgrounds. Moreover, it refrains from making predictions when uncertainties arise, instead identifying the need for human intervention, so combining automation with human in-the-loop verification [50].

One impact of using automated species identification is that it opens up citizen science participation to a more diverse set of participants. For instance, a comparison of two similar citizen science projects focusing on mosquitoes—one analogue, where participants submit physical samples by post, and one digital, where participants submit photos through an app—showed that there was a significantly higher proportion of female, younger and non-academic participants in the digital project [51]. Also, in 2020, BirdNET—an app that uses artificial intelligence to identify birds based on sound—engaged more than 1.1 million participants compared to the 317,792 participants of eBird—an app where participation requires pre-existing identification skills [52]. While BirdNET generates probabilistic, less well-validated data, it may act as a gateway for participants to become skilled birders who may then move on to the more advanced protocols of eBird. Although acoustic monitoring is not yet routinely deployed for insects, bush crickets have been monitored as part of a citizen science bat monitoring scheme in France since 2006, which has resulted in 16 349 individual sampling locations and the detection of significant declines for several species [53,54]. Recently, scientists have also been able to distinguish European honeybees from wild bees based on wing beat signatures [55,56], and it is possible that future monitoring of pollinators could be done by farmers or gardeners with small audio or movement recorders [57,58].

## 5. Technology as an enabler—opening up new research avenues

Besides facilitating existing citizen science projects, technology also offers the promise of enabling new ways of doing citizen science. One fruitful opportunity is through more complete ecosystem monitoring, rather than reliance only on popular insect groups as bioindicators [59]. The use of emerging technological equipment can enable the monitoring of species that are small, elusive, cryptic or hard to identify and simultaneously open people's eyes to nature that would otherwise be inaccessible to them [60]. Molecular methods, such as environmental DNA (eDNA) sampling, continuous real-time observation by cameras, audio recorders and remote sensing using drones, lidar and laser vibrometry [61] can help detect the diversity, behaviour and interactions of insect species overlooked by the human eye and ear. Technologies, such as infrared sensors, audio sensors and image-based classification, that were developed for pest management [62] show that it is possible to count and differentiate insects as small as aphids and fruit flies at least to order level [63–65]. Sensitive acoustic recorders have been used to detect non-vocalizing beetles based on sounds made from chewing, biting and movement activities [66–68] and so could be developed for use in citizen science.

Technologies can also be used in citizen science activities to fill in spatial or temporal data gaps by increasing observations in remote regions or at unsociable hours. For instance, the monitoring of nocturnal bird migrations with acoustic recorders became popular in the United Kingdom during the COVID-19 lockdowns. The process involved the use of sound recording equipment and computer software for call signature identification and has led to build-up of online communities through which to share expertise, e.g. xeno-canto [69]. Similar advances enabling citizen scientists to use technology to fill spatial and temporal gaps could be explored for different types of insect monitoring; for example, camera traps for automatic monitoring of night-active insects are being developed. The advances are rapid—just a couple of years ago they could only identify few and distinct species [33,70], whereas now automated moth identification can be done for thousands of species [71].

Collection of samples for DNA analysis is an enticing and valuable future prospect for involving citizen scientists in expedited biodiversity assessments by collecting DNA samples from the environment, such as from soil, water, plants and air, instead of the insects themselves [72–74]. DNA sampling and analysis of single individuals or bulk insect samples are being developed rapidly and have already been implemented in citizen science projects, where participants have collected bulk insect samples in nets fixed above their cars (e.g. [75,76]). These collection methods could be further combined with recent developments in robotics, where systems such as the DiversityScanner [77] and the BIODISCOVER machine [33] can help speed up the processing of the many bulk samples collected by citizen scientists.

Environmental DNA sampling from water, soil, plants, faeces and air goes one step further by not necessitating species' presence when sampling [78] and eDNA metabarcoding has demonstrated notable efficacy in the detection and surveillance of terrestrial and semi-aquatic animals (e.g. [73,79]). This may prove especially important due to recent increased focus on ethical insect monitoring and the shifting public opinion of insects [80]). Importantly, it has been shown that citizen scientists with minimal training are effective in eDNA sampling [72], thus opening participation to people who have previously been under-served, and participants can even be involved in the corresponding laboratory analyses [81,82]. Furthermore, such participation can enhance citizen scientists' understanding of biodiversity, ecosystems, and the principles of eDNA [73,83]. A further step could be the development of commercially available in-field diagnostic tests, similar to the COVID-19 lateral flow tests, which could be developed for rare or invasive species [74,84].

At the cutting edge of technological development, lidar has been employed to monitor patterns of insect swarms around the top of wind turbines [85], while radar can track aerial movements of e.g. birds, bats and insects such as ladybirds [86–88]. Most recently, photonic sensors have been shown capable of distinguishing 30 free-flying hoverfly species and their sex by spectral analysis of thin-film wing interference signals [89]. Many modern cars are fitted with high-resolution cameras, lidar and radar systems that are capable of detecting insects. Conceivably, there is future potential for a global network of millions of cars counting insects [90], although the provision of feedback to public participants will be crucial in evolving this into engaging citizen science (rather than just extracting data from sensors). Already, researchers have used Google Street View images to map the distribution of insects such as the pine processionary moth (*Thaumetopoea pityocampa*) from its easily detectable larval nests in pine trees [91], and citizen science annotations provide crucial datasets for training the algorithms [92]. Lidar is now also included within some premium smartphones, and could potentially be used for assessments of habitat condition [93,94]. This demonstrates how expensive technology can quickly be miniaturized and made affordable and accessible by technology companies, just as happened when GPS was included as standard in smartphones 15 years ago. If, in the future, platforms like iNaturalist were to incorporate the capability to upload lidar scans of the habitat where a species was observed then scientists would have a detailed, yet objective, description of the habitat in which a species occurs [94]. These scans could be further analysed and annotated using crowd-sourcing platforms such as Zooniverse.

Technology also offers solutions to tackle one of the biggest critiques of citizen science: data quality [95–97]. Manually verifying the increasing amounts of records is time-consuming, error-prone, difficult to reproduce and limited to known geographical areas and taxonomic groups. Some data platforms, such as iRecord, Artsobservasjoner and Observation.org, include plausibility checks based on predefined rules [98]. However, more sophisticated artificial intelligence algorithms could be developed to perform automatic checks of submitted data, e.g. plausibility of the record based on existing data or image classification of uploaded photos, as already included in some of these platforms, and so provide immediate feedback to data providers [28,99–101]. In addition, natural language generation [102] and real-time feedback to volunteers could aid species identification. For example, upon submission of a photo and suggested species name, feedback can be sent explaining reasons for misidentification and highlighting key features to look for in order to identify the species correctly, enabling the participant to learn and improve over time.

Technology can also be used to directly influence recorders. Existing recorders can be informed where best to record based on current data [103] (and see also the DECIDE tool in [104]). There has also been exploration of the potential to implement chatbots in citizen science projects or on social media. Project participants or social media users can be prompted to provide more information or to look for other species, such as host plants or things like habitat and environmental conditions [97,101].

New technologies can support learning in other ways as well. In addition to easy access to learning materials, such as videos and tutorials (see Box 1), social interaction on apps or websites can also foster social recognition, visibility of contributions and reputation gain through regular feedback from the citizen science project and—possibly combined with motivational designs using game-like elements—can enhance the participation and retention of citizen scientists by rewarding participants with visualization of personal activity and achievements [105].

## 6. Technology as a transformer—rethinking science and collaboration

The inclusion of technology in citizen science may transform the way that people think about science and nature, leading to a fundamental reorganization of the monitoring landscape of actors and activities [106]. Monitoring our living world is not done solely by academic researchers or experienced naturalists but, when supported by the use of new technologies, can be done by anyone who has an interest, regardless of expertise. By changing the types of tasks and necessary skills, technologies can engage participants with diverse backgrounds, promoting inclusivity and widening societal engagement [51]. The development and inclusion of affordable technology in citizen science may help fill geographical data gaps, which is a major limiting factor of current biodiversity databases and large-scale predictions of biodiversity trends [97]. It could also help increase the reach of citizen science in the global south and make citizen science more inclusive [107,108]. Online platforms, such as Zooniverse and Agouti [109], are enabling citizen scientists to explore the natural world virtually through sounds, photos and images, can transport people beyond the places they can physically explore and generate new interest in the natural world [110]. Platforms like iNaturalist and CitSci allow participants to create their own projects, democratizing science and empowering people to influence and drive change [36].

By combining technologies, we may further expand what is possible. The KInsecta project is developing a platform designed to automate observation and identification of pollinating insects through combining a variety of sensors, including automatic image recognition and the precise measurement of wing beats. The entire sensor system is designed to be accessible to citizen science enthusiasts, allowing them to build and operate the hardware independently at a minimal cost [111]. Automated multisensor stations are being conceived for combined monitoring of multiple aspects of biodiversity. Current iterations include automatized visual monitoring, image analyses and bioacoustics monitoring [71], but could be extended to the detection of smellscapes using volatile organic compounds or malaise and pollen traps for metabarcoding [70].

The prospect of real-time whole-ecosystem monitoring—when there are seamless data flows between collection and analysis—has broad repercussions. Real-time interaction and prompts may increase the information content of collected data and the experience and knowledge gained by citizen scientists. By combining data streams with artificial intelligence, projects like BirdCast and Whale Safe are guiding people to take action that optimally benefits nature by turning off lights at night or reducing ship speed, respectively, to protect migrating animals at critical times and places [112,113]. Connecting live streams leads to the development of the ‘Internet of animals’, which is currently mostly employed for vertebrates [90]. Such real-time monitoring also contributes to the development of digital twins of landscapes, which comprise statistical and mechanistic models that are continuously calibrated with real-world data [114]. Digital twins are attracting attention across the environmental sciences for their potential in improving our understanding of ecosystems and also in supporting decision-making about contrasting policy and management options. Improved predictive models and forecasting could help identify the general pathways towards ‘bending the curve’ of species loss [115] as well as help to tackle specific problems such as predicting pest outbreaks or the spread of insect-transmitted diseases.

## 7. Technology as an inhibitor—complicating methods and excluding participants

The strength of citizen science projects lies in their participants and potential for collecting and/or analysing large amounts of data. Although we have discussed the positive role of technology in facilitating, enabling, and even transforming citizen science, it is also possible that technology can act as an inhibitor by limiting people's involvement with and connection to nature and by increasing costs, both for equipment and for the staff needed for data processing, and prolonging verification times. Furthermore, many technologies are in a developmental stage and ceding authority to them prematurely may lead to an increase in inaccurate or biased data. It is easy to get excited by new technologies, but just because we can implement them in citizen science activities does not mean that we should.

Traditional citizen science monitoring projects, especially for insects, are generally biased towards older generations and men [116–118], which may affect rates of uptake of new technologies. Current participants may disengage if the deployment of technology restricts the range of potential contributions that they can make, resulting in tasks that are either overly simplistic or excessively complex [119]. Technology can create a barrier between people and nature, increasing people's distance from nature or the sense of commodification of nature, thus reducing nature connectedness [120]. This can happen either through the phone screen acting as a filter through which people experience nature, or by reducing people's direct engagement, e.g. just taking water samples for eDNA analysis rather than searching for and directly observing organisms. Other deterring factors may include lack of acknowledgement [121], a diminished sense of community such as that reported by students with online teaching compared with face-to-face teaching [122], and uncertainties about privacy and data protection, especially if citizen science projects start tracking the movement of participants in order to estimate observation effort.

**Figure 4.**
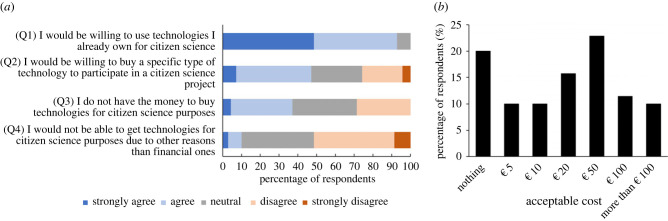
(*a*) Survey answers to questions regarding willingness to acquire or use pre-owned technologies for citizen science projects. (*b*) Survey answers to the question ‘How much would you be willing to pay to participate in citizen science?’. Number of responses given in percent out of 70 respondents in total.

It is also likely that we will see an initial further increase in the biases of data collection towards countries from the Global North where technologies are being developed and likely to be deployed first [119]. Implementation of technology in citizen science projects could be prohibitive for participants who are unable to access or use the necessary technology [123]. For example, in a survey of 27 countries, national smartphone ownership among adults ranged from 47–98% between countries, while Internet usage varied from 56–99% [124]. Furthermore, technology compatibility issues may also inhibit participants' involvement in a project; for example, LeafByte, a citizen science app for measuring the area of leaves [125] and Monarch SOS, an app for identifying and recording monarch butterflies [126], are currently only compatible with Apple's proprietary iOS platforms. Updates to smartphone operating systems (Android or iOS) can make apps inoperative until they are updated, at cost to the project organizer. Where this inability to access the necessary technology is largely financial, it may become less problematic as technologies develop and become cheaper. Respondents in our survey (mainly citizen science organizers) did show some willingness to buy equipment specifically to participate in citizen science projects (figure 4*a* Q2), but the amount that they were willing to spend varied (figure 4*b*) and participants were more inclined to use equipment they already own and control (figure 4*a* Q1). Accessibility of technology is therefore an important consideration when developing new citizen science projects, and this is especially true for indigenous people for whom technology may be especially inaccessible, yet whose territories cover 22% of the world's land surface and 80% of the world's biodiversity [127]. Researchers working in partnership with these people should consider costing the provision of technology into funding proposals to reduce these barriers, and should adapt applications to serve local issues, e.g. using pictures rather than words in apps when working with non-literate people, as was done in the Sapelli collector [128].

It is also important to consider the sustainability of access to these technologies once implemented. Despite increasingly available low-cost, user-friendly technology, there can be huge costs in developing platforms for insect citizen science and maintaining their long-term viability, stability and security, especially those that serve thousands of participants. These include incurring initial implementation costs (associated with the acquisition and installation of technological infrastructure), ongoing maintenance costs (for the technical support to ensure full functionality, and expenses for regular maintenance updates), operational costs (such as the day-to-day expense of using technology, for example energy consumption and licensing fees) and integration costs (if the new technology needs to be integrated within existing systems to ensure seamless compatibility and data transfer).

Bias in data collection may unintentionally lead to a bias in technology performance, especially because image classification algorithms will be influenced by the data on which they are trained [129]. For example, most iNaturalist users rarely record the same species twice [130] and rarities are reported and documented more often than common species [129], which can furthermore create major issues in using the data for ecological monitoring. In the case of smartphones, technology is currently a limitation for capturing images of small or fast-moving insects due to limited camera resolution, focus distance or shutter speed [131]. Some within-camera software can create visual artefacts that would affect identification accuracy. This is likely to enhance people's natural bias towards large and conspicuous species, although camera hardware and software in smartphones may improve over time to overcome these limitations. However, as hardware and AI algorithms advance over time, technology could lead to a reverse shifting baseline enabling detection of previously overlooked species, so researchers will need to consider this when seeking to use their data for long-term insect monitoring.

Scientists also need to consider the ethical challenges associated with collecting, storing and sharing sensitive data, like movement patterns of observers or locations of endangered species. Although there is much advice on this topic (e.g. [132]), automation of data capture and publishing could lead to unforeseen risks that should be carefully considered in advance. Another ethical issue concerns the ownership of data gathered and analysed by citizen scientists; for example, there might be ambiguity over the intellectual property rights for novel data/pictures of species new to science [26]. To pre-empt such disputes, a written code of conduct that defines the necessary procedures, including good data citation practices, may be appropriate. Platforms need to make clear which data are being collected, the meanings of different licences (e.g. which of the six Creative Commons licences should apply; see https://creativecommons.org/share-your-work/cclicenses/) and the potential risks and possible downstream uses of data that are shared (e.g. that images being shared may be used to train machine-learning algorithms).

Another ethical challenge is that many emerging technologies used in citizen science are closed source (i.e. proprietary), meaning they are legally enforced ‘black boxes’ preventing users from studying how they work or adapting them to suit different needs. Proprietary technology enforces lock-in to a single vendor, high switching costs and lack of interoperability and reproducibility. This is especially pertinent to citizen science, where many participants are doing science for the first time, and using proprietary technologies may inadvertently normalize a 'black box' approach to science. Therefore, as emphasized in the UNESCO Recommendation on Open Science [133], open source technologies—defined as those with freedoms for users to use, study, modify and share them without restrictions [134,135]—are essential for not only economic savings [136] but also more inclusive and equitable research, especially outside the Global North (e.g. [137,138]). Successful examples of open innovation in citizen science include the source code of the iNaturalist mobile apps or hardware designs for the EnviroDIY water quality sensors, while successful business models have been developed to support them, such as the company Open Acoustic Devices for acoustic ecological monitoring [139]. Citizen science practitioners should exercise due diligence in searching for, adopting and developing existing open-source technologies in their projects.

Finally, the possibilities of emerging technologies should not lead to the assumption, both by coordinators, volunteers and especially funders, that all citizen science projects need to employ or advance technology. As discussed throughout this manuscript, technology should not be seen as a panacea, and it can also place additional burdens on coordinating scientists because they may not be experts in data science, machine learning, or high-performance computing. As a solution, effective collaborations should be sought, bringing together diverse expertise from ecologists and taxonomists to data scientists and IT specialists (see the PRISE project [140]) to help citizen science projects to be designed as fit for purpose, and then these consortia also need adequate funding for these collaborations and technology development.

## 8. The future is bright and should be diverse

The future of citizen science and insect monitoring, enhanced by technology, presents promising prospects for research advances and participant engagement and raises critical questions about equity. Scientists and project organizers should look to the future for the benefits that new technologies can bring to citizen science, but should be careful to avoid the trap of inflated expectations of these new technologies. First and foremost, technology offers the opportunity to work towards global, whole-ecosystem monitoring, including small, cryptic and elusive insects coupled with species behaviour, movement and interactions. Technology can serve as a powerful tool to extend and democratize data collection, processing and validation, but raises concerns about exacerbating existing disparities. Bridging this gap will require thoughtful design of projects and implementation, considering the local infrastructure, technological literacy and available resources of the potential participants. While technologies are constantly developing and becoming cheaper and attainable for more people, it is important that their use is context-appropriate rather than their inclusion being solely for the sake of their novelty. Participatory development of the technologies [36] can help to align needs and empower citizen scientists to find joint solutions for participant engagement in citizen science [105]. This shift toward inclusivity and appropriate use of technologies should prioritize regions with limited access to scientific resources, fostering global collaboration and supporting data collection in some of the data-poorest areas.

The responsibility for creating an equitable future for technology-driven citizen science lies with both coordinators and participants. Coordinators should ensure that their applications are user-friendly, open source, compatible with various operating systems and open to diverse participants, with multiple access points allowing for flexibility in what the participants wish to learn [141]. For example, iNaturalist and Pl@ntNet have been globally successful in large part because of their customizability, allowing the inclusion of place-based localized projects within the greater platform ecosystem (e.g. [142]).

The ongoing value of skilled citizen science participants making field observations without technological devices should also still be recognized. Engaging new participants should not come at the cost of disengaging previous ones. We strongly advocate that advances in citizen science monitoring of insects with new technologies should seek complementarities and diversification rather than replacement. Long-term monitoring schemes, with continuity of methods, are essential in providing consistent evidence for decision-making so researchers need to consider how new technology can be incorporated to support, not scupper, the consistency and longevity of this monitoring. Fortunately, new statistical approaches can help. Integrated distribution models [143,144] allow information from multiple data streams (e.g. traditional citizen science and new technologies) to be harnessed. Such models can also include the probabilistic data collected through autonomous sensors (e.g. camera traps and acoustic recorders) and analysed using deep learning algorithms [145].

New technologies provide opportunities to enhance insect monitoring through citizen science in so many ways: they can facilitate and make easier what is already done; they can expand the potential of citizen science to contribute to new monitoring; and they can help to transform the relationships between people and nature, and between different communities of people, to enhance equity and diversity in our environmental monitoring. Here, we have focused on the current state and development of 11 technologies, some of which have already seen wide implementation while others are in their infancy, but we deem show great scientific potential (table 1). While not all technologies have been implemented in citizen science projects for insect monitoring, we believe there is much to be excited about. As we navigate the increased technology and capabilities in environmental and insect monitoring it is essential that we collectively strive to make technology-enhanced citizen science an avenue where diversity and participant engagement are at the forefront of our efforts. In this way, emerging technologies can truly foster and enhance the engagement and impact of citizen science.

**Table 1.** Examples of how the technologies presented in the online survey are being used in citizen science projects. Their current level of implementation in citizen science projects (implementation), level of engagement between people (engagement) and untapped scientific potential (potential) were independently scored from 0–3 where 3 is the highest by authors J.K.S., T.A., D.E.B. and C.T.C. and a consensus reached.

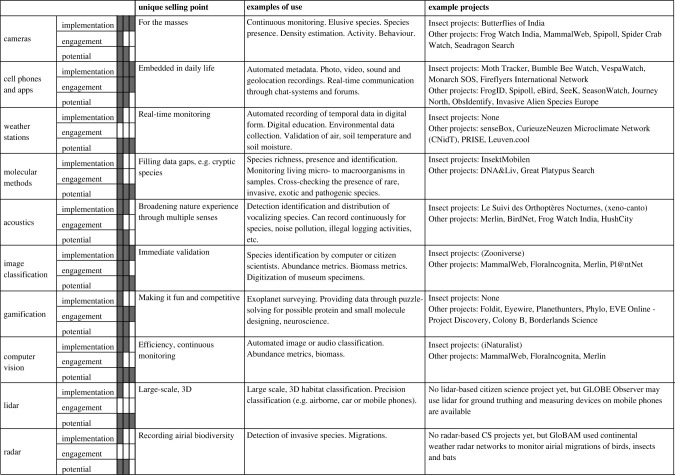

## Data Availability

The data are provided in Zenodo [24] and electronic supplementary material [146].
